# The Effect of Concave-Side Intertransverse Ligament Laxity on the Stress of AIS Lumbar Spine Based on Finite Element Method

**DOI:** 10.3390/bioengineering9120724

**Published:** 2022-11-23

**Authors:** Linjie Zhang, Qiaolin Zhang, Yan Zhang, Musinguzi Arthur, Ee-Chon Teo, István Bíró, Yaodong Gu

**Affiliations:** 1 Faculty of Sports Science, Ningbo University, Ningbo 315211, China; 2 School of Chemical and Biomedical Engineering, Nanyang Technological University, Singapore 639798, Singapore; 3 Faculty of Engineering, University of Szeged, 6720 Szeged, Hungary

**Keywords:** intertransverse ligament, ligament relaxation, adolescent idiopathic scoliosis, finite element, stress distribution

## Abstract

**Simple Summary:**

An effective finite element model of the lumbar spine was established to explore the effect of concave-side ITL laxity on the biomechanical properties of AIS. It was found that concave-side ITL laxity could reduce the stability of the lumbar spine, aggravate the uneven stress distribution of lumbar scoliosis, increase the risk of lumbar disc injury, and have an adverse effect on the lumbar spine with scoliosis. Simply releasing concave-side ITL is not an effective method for the treatment of scoliosis.

**Abstract:**

(1) Background: Scoliosis has the mechanical characteristic of asymmetric stress distribution, which is one of the reasons for the aggravation of scoliosis. Bracing therapy is the best treatment for AIS, but it is difficult and costly to operate. Is it possible to reduce pressure in the concave side by relaxing the ITL in the concave side of scoliosis, so as to improve the abnormal stress distribution of scoliosis? In this paper, a finite element method was used to simulate the effect of the relaxation of concave-side ITL on the stress of a lumbar spine with scoliosis, which provides some guidance for the treatment of scoliosis. (2) Methods: Using CT images of a patient with scoliosis whose Cobb Angle was 43° and Lordosis Angle was 45, a scoliosis lumbar was established, and Young’s modulus of the ITL of the concave-side lumbar spine was reduced by 95% to simulate ligament relaxation. By comparing the stress condition of the model vertebral body with no ligament relaxation, the effect of concave-side ITL relaxation on the mechanical characteristics of scoliosis lumbar spine was explored. (3) Results: An effective and complete model of the lumbar spine was established. The concave ITL relaxed, which only had a great impact on the bending loads. After the ligament was relaxed, the stability of the spine was reduced. Stress concentration on the concave side of vertebrae and the IVD was aggravated. Under loads on the convex side, the maximum stress on the vertebral body and the IVD increased significantly, making lumbar vertebrae more vulnerable to injury. (4) Conclusions: Laxity of the ITL on the concave side of the AIS lumbar only affects the bending load. Laxity of the concave-side ligament will reduce the stability of the lumbar, aggravate the uneven stress distribution of scoliotic lumbar vertebrae, increase the risk of IVD injury, and be unfavorable for the scoliotic lumbar spine. Relaxation of the concave ITL alone is not an effective way to treat scoliosis.

## 1. Introduction

Spinal dysplasia is a common juvenile disease. Adolescent idiopathic scoliosis (AIS) is a spinal disease with high incidence; most patients are 10–16 years old [1]. It can cause a variety of three-dimensional deformities of the spine but is also accompanied by lower back pain, decreased cardiopulmonary function, and other symptoms [2]. The etiology of AIS is not clear [3]. Some studies have shown that uneven force on the vertebral body may be one of the causes of scoliosis [4]. Meanwhile, ligament relaxation is also one of the clinical manifestations of AIS and spinal overgrowth [5]. The treatment of scoliosis is also complicated, and the effect of surgical treatment and non-operative treatment represented by a brace is still controversial [6]. The treatment of scoliosis usually involves support and surgical instruments. Support management is an effective means of treating AIS [7]. The mechanism of the scoliosis treatment device is to generate support for the scoliosis spine and stress on the convex side. Hueter et al. [8] pointed out that increasing stress will stop bone growth; whereas, reducing stress will accelerate bone growth, so reducing the pressure on the concave side of scoliosis can slow down the progress of deformity in the process of growth [9]. The intertransverse ligament (ITL) is the ligament connecting the transverse process, which is located on both sides of the vertebrae and limits the excessive bending of spine to opposite side [10]. Due to the abnormal physiological morphology of scoliosis, the mechanical effect of ITL on the scoliosis spine is also changed. The finite element (FE) method has been used to explore whether uneven stress distribution of the vertebral body can be reduced by relaxing the concave-side ITL to slow down the progress of deformity.

The pathogenesis of scoliosis is not clear. However, there is an obvious clinical phenomenon in AIS patients that the anterior vertebrae of the spine grows faster than the posterior vertebrae [11]. However, anterior growth of the spine is also a clinical manifestation in patients with AIS. Overgrowth in the front of the vertebrae can disrupt the biomechanical properties of the lumbar, leading to spinal instability and abnormal morphological development [11]. Other studies have shown that relative anterior spinal overgrowth is the result of AIS [12,13]. However, relative anterior spinal overgrowth is a clinical phenomenon that occurs in AIS patients. Some hypotheses have used this phenomenon to understand AIS etiology. Based on this phenomenon, Cheng [14] established a 3D spring model to explore the role of the ligament in the mechanism of scoliosis. The ligament is important in the progression of AIS. There are 23% of AIS patients that also suffer from fibrin metabolism disorder, which makes the cells unable to combine with external fibrin. This indicates that the elastic fiber system of the ligament plays a potential role in the pathogenesis of AIS [15].

Due to the limitations of experimental conditions in in vivo experiments, it is difficult to explore the effect of ligament stiffness on the biomechanical properties of the lumbar [16]. The FE method can simulate the motion of the lumbar, and the motion states of the spine can be simulated by load and boundary conditions [17], to explore the mechanical characteristics of the lumbar: stress distribution, strain, displacement and so on. In 1973, Belytschko et al. [18] used the FE method in the field of spinal research for the first time [19]. Zhang et al. [20] established scoliosis lumbar vertebrae and analyzed the abnormal stress characteristics of scoliosis lumbar vertebrae under different loads. Kamal et al. [21] used FE analysis to explore stress changes of the lumbar endplate in the upright state after paralyzing unilateral muscles. This study found that the paralyzed concave muscles can alleviate the uneven stress distribution of the vertebral body, thus slowing down the progress of deformity. In previous studies, there are few studies on relaxing the ITL ligament to reduce vertebral pressure. This paper established an FE model of an AIS lumbar, and six different loads were applied to simulate lumbar flexion, extension, left and right lateral bending (LLB, RLB), and left and right axial rotation (LAE and RAR). The stress of lumbar vertebrae without concave ligament relaxation was compared with that of relaxed lumbar vertebrae to explore the effect of concave ITL relaxation on the stress of AIS lumbar vertebrae. The purpose of this study was to explore the effect of concave ligament relaxation on scoliosis lumbar stress and to explore whether it is possible to relax the ITL of the concave-side to alleviate the uneven stress distribution of the vertebral body and improve ordinary AIS treatment.

## 2. Materials and Methods

### 2.1. Data Acquisition

The subject of paper is a 15-year-old woman with congenital scoliosis, weighing 45 kg. The Cobb angle of the lumbar spine is 43° and lordosis angle is 45°. The subject was in a supine position without weight. The subject was CT scanned (Siemens, Munich, Germany), the scanning interval used was 0.75 mm, and the CT image was converted into DOCM format in Mimics (Materialise, Leuven, Belgium) for follow-up 3D modeling [22]. This study was approved by the Ethics Committee of Ningbo University (RAGH20220321), and the subject fully understands the purpose of the experiment.

### 2.2. Establishment of the Model

The 2D CT image was imported into the medical imaging software Mimics 20.0 (Materialise, Leuven, Belgium) and extracted as a 3D model, then the model was imported into Geomagic Studio 2013 (Geomagic, Inc., Research Triangle Park, NC, USA) to smooth out the model, then the model was imported into SolidWorks 2020 (SolidWorks Corporation, Waltham, MA, USA) for model assembly. At the same time, the anatomical structure of the intervertebral D (IVD), endplate and articular cartilage was established.

### 2.3. Material Parameters

In this study, cancellous bone, cortical bone, articular cartilage, endplate, and IVD are isotropic uniform linear elastic materials [23,24]. Their material properties are shown in Table 1. The lumbar vertebrae are composed of a cortical bone surrounding cancellous bone. It has been found that the thickness of vertebral cortical bone ranges from 0.2 mm to 2 mm [25]. The cortical bone thickness used was 1 mm. The endplate thickness was 0.8 mm. The contact property of the facet joint surface was friction contact. The connection mode between articular processes was set as friction [26].

The established model was imported into ANSYS Workbench (ANSYS, Inc., Canonsburg, PA, USA), and a mesh sensitivity test was carried out before analysis [27]. The material properties and the mesh size are shown in Table 1 [28].

In this study, seven kinds of major ligaments, namely, the anterior and posterior longitudinal ligament, the interspinous ligament, the capsular ligament, the flaval ligament, ITL, and the suspraspinous ligament were simulated using a line element with tension only. In this study, line elements were inserted into the cone surface according to the anatomical structure, and the material parameters of various tissues were taken from the literature [4,28,29,30,31]; the ligament material parameters are shown in Table 2. According to Kamal et al. [21], the experimental data related to a 95% loss of strength in rabbits’ BTX-A injected muscles [32] simulated muscle weakness by reducing the muscle physiological cross-sectional areas of the concave target longissimus thoracis pars thoracic and multifidus lumborum muscles. Due to a lack of related research on ITL relaxation, this study also reduced Young’s modulus of the concave ITL by 95% to simulate ligament relaxation.

### 2.4. Boundary and Loading Conditions

In this study, the finite element model of lumbar vertebrae was statically analyzed. The boundary and load were employed using Yamamoto’s [33] in vitro test loads fixed on the lower side of the L5 vertebrae to restrict its range of motion in all directions and a numerical downward load was applied on the upper surface of the L1 to simulate human body weight. The lumbar spine bears about two thirds of the human body weight, so the load used was 300 N and the torque of 10 Nm [34] was applied to simulate six different states of lumbar flexion, extension, LLB, RLB, LAR, and RAR. The stress of the model vertebrae, IVD, and ROM of joints were observed.

### 2.5. Model Validation

Because there is no accepted verification standard for different angles of scoliosis [35], model verification was used to verify whether the modeling process was correct or not. Therefore, if the normal lumbar model is verified to be effective, the scoliosis lumbar model is also effective [36]. By comparing the established model with the in vitro experiment, the stiffness of the lumbar is compared to verify the validation of model.

*K* = *M*/*θ*

In the formula: *K* is the average stiffness, *M* is the applied moment, *θ* is the angular displacement. The normal FE lumbar model is compared with the in vitro experiment.

## 3. Results

### 3.1. Established Model

**Figure 1:**
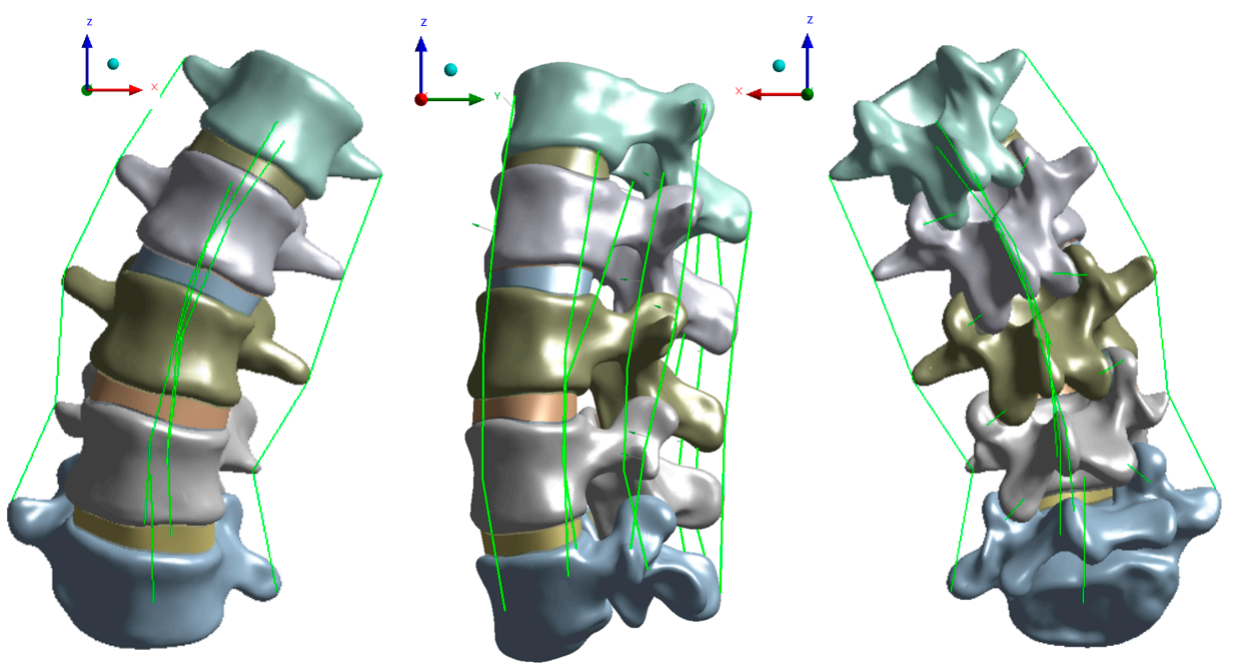
3D model and ligament of scoliosis lumbar vertebra.

A complete L1–L5 lumbar spine model was established, including 335,214 tetrahedral solid elements and 581,618 nodes. The model is shown in Figure 1. The model includes cancellous bone, endplate, annulus fibrosis, articular cartilage, and nucleus pulposus. The anterior longitudinal ligament, posterior longitudinal ligament, ligamentum flavum, interspinous ligament, capsular ligament, supraspinous ligament, and intertransverse ligament was established. The material property of the ligament is the line unit with only tension. The established model was compared with the in vitro experiment to prove the effectiveness of the model.

### 3.2. Validation Results

The stiffness of the model was recorded by applying the pure moment of 10 NM. The moment applied on the upper surface of L1 and the stiffness result is shown in Table 3 [37]. Compared with the in vitro experiment, the stiffness of the model established in this study is slightly different from that of the in vitro experiment except for the extension load; whereas, Yamamoto’s in vitro experiment is a normal lumbar experiment. The model established in this paper is a scoliosis lumbar model. Because there is no data on the stiffness of the cadaver experiment of scoliosis lumbar, and the stiffness of lumbar vertebrae with different degrees of scoliosis is also different, this paper can only compare with that of normal lumbar vertebrae. At the same time, under the extension load, the stiffness of the model is quite different from that of the in vitro experiment.

### 3.3. Lumbar Range of Motion

**Figure 2:**
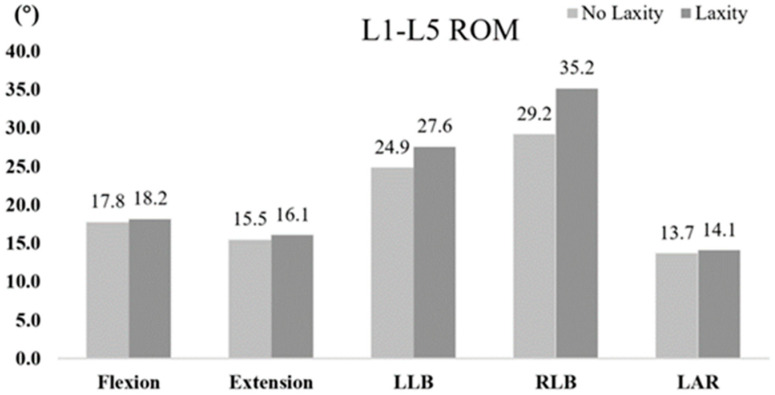
Range of motion of lumbar vertebrae.

After ligament relaxation, the ROM of lumbar vertebrae increased under all loads, but the ROM of L1-L5 was more obvious under the lateral bending load, with an increase of 10.8% in the left bend and 21.4% in the right bend, as shown in Figure 2. In this study, the ITL on the concave side, that is, the left side, was relaxed, resulting in a large increase in the ROM of the lumbar spine during the right bend. After the relaxation of the concave ligaments of scoliosis, the stability of the lumbar spine decreased.

### 3.4. Vertebral Stress

**Figure 3:**
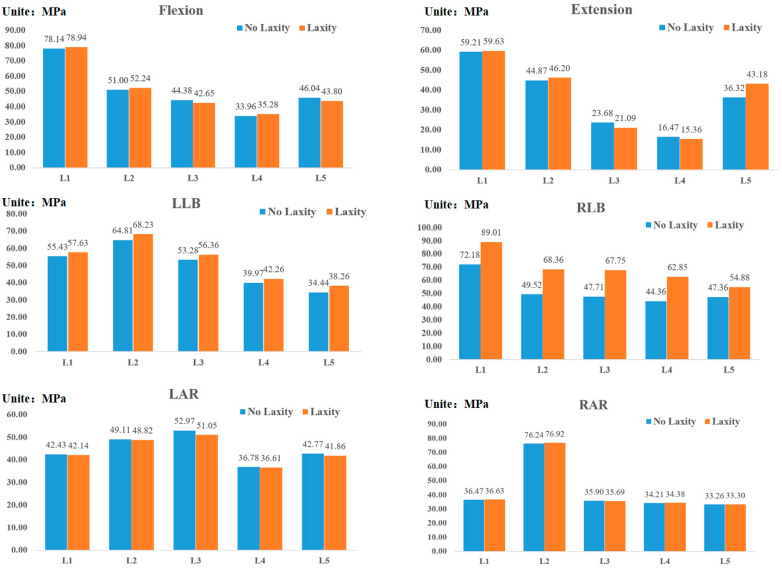
The maximum stress value of the vertebral body under each load.

The same load was applied to the lumbar vertebrae with loose concave ligaments and the unrelaxed lumbar vertebrae, and the maximum stress on the vertebrae is shown in Figure 3. After relaxation of concave ITL, only the bending load was affected. Under the LLB (concave bending) load, the stress vertebral body of L1–L5 increased slightly to 3.9%, 5.3%, 5.8%, 5.8%, 5.7%, and 11.1%, respectively. Under the RLB (convex bending) load, the maximum stress of vertebrae of L1-L5 increased greatly. The maximum stress of L1~L5 increased by 23.4%, 38.4%, 42.0%, 41.7% and 15.9%, respectively.

**Figure 4:**
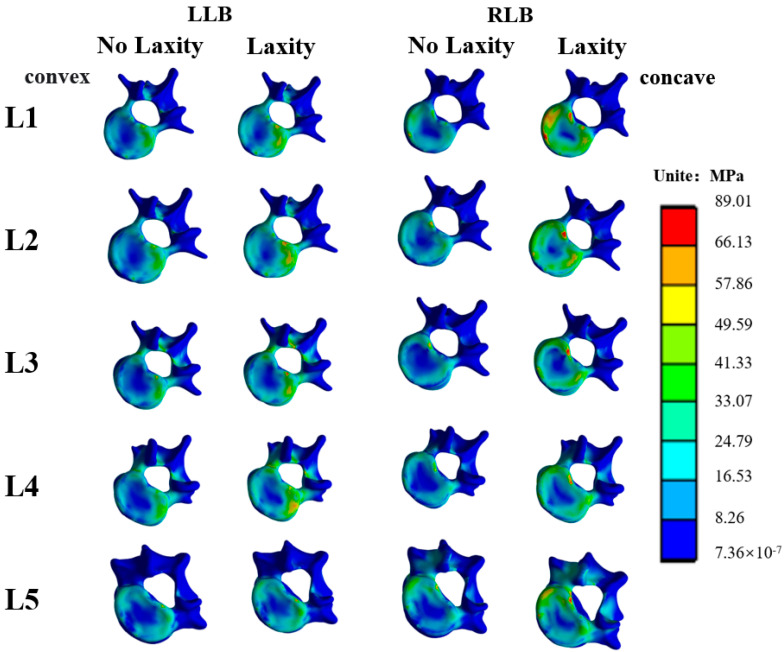
Stress distribution of vertebral body under lateral bending load.

Under loads of lateral bending, it can be seen that under the LLB, the stress on the concave side of vertebrae tends to concentrate after relaxation of the ITL on the concave side, as shown in Figure 4. Under the RLB, the stress on the convex side of vertebrae is more concentrated after the ligament is relaxed.

### 3.5. Intervertebral Disc Stress

**Figure 5:**
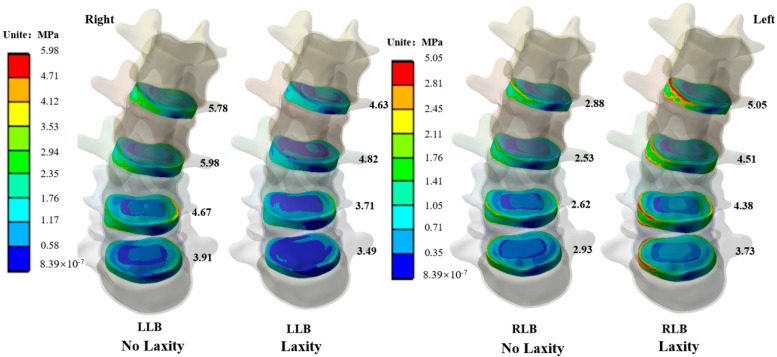
Stress distribution of IVD under lateral bending load.

After the relaxation of concave-side ITL, the ROM of the lumbar under the lateral bending load is the most affected, so the stress value and stress distribution of the IVD under the lateral bending load have been explored. As shown in Figure 5, under the LLB load, after the ligament relaxed, the maximum stress of IVD increased, and the stress of IVD increased by 24.8%, 24.1%, 25.9%, and 12.0%, respectively. Under the RLB load, after the ligament was relaxed, the maximum stress value of the IVD increased, and IVD stress value increased by 75.3% 78.2% 67.2% 23.3% from up to down, the stress on the convex side of IVD increased significantly. After relaxation of the concave ligament, the stress change trend of IVD in the lateral bending load was similar to that of the vertebral body, but under the RLB after ligament relaxation, the stress of IVD increased more obviously.

## 4. Discussion

In this study, according to the lumbar CT image of an AIS patient whose Cobb angle of lumbar spine is 43° and lordosis angle is 45°, we used relevant modeling software to convert 2D imaging data into a solid 3D model. The Cobb angle and lordosis angle of the model are consistent with the results of the 2D imaging measurements and will not change. The stiffness of the established model is consistent with the in vitro experiment. Therefore, the model is valid. Young’s Modulus of ITL on the concave side was reduced by 95% to simulate ligament relaxation to explore the effects of concave ITL relaxation on lumbar joint motion and stress on vertebral body and IVD. It is complicated to treat scoliosis. There are few studies on the ligament stiffness of scoliosis lumbar vertebrae. The FE method can be used to explore the effect of concave ITL relaxation on lumbar mechanics. It was found that after the relaxation of the ITL of the concave side, the ROM was increased, especially under the lateral bending load. After the ligament relaxation, the stress on vertebrae and the concave side of IVD will increase when bending to the concave side. When bending to the convex side, stress on the concave side will also increase, especially on the concave side of the IVD. After ITL relaxation in the concave side, the stability of the lumbar spine is reduced, and the stress of the vertebral body is further increased. In this environment, scoliosis will be further worsened. At the same time, the pressure of IVD is increased, which will lead to the incidence of IVD disease.

The FE method has significant advantages over in vivo experiments [39]. It can achieve the effect of an in vivo experiment which is difficult to achieve through computer simulation technology without violating ethics [40]. In the spinal FE analysis, various motion states of the spine can be simulated by changing the load and boundary conditions, and the pathological changes of the spine can be simulated by changing material properties of model [22]. In the field of spinal research, the FE method is often used to analyze the stress of the spinal brace and to explore etiology. The treatment of AIS is complicated, and the effect of surgical treatment and non-operative treatment represented by the brace is still controversial [3]. Given the above situation, the FE biomechanical method has been widely used in the treatment and etiological study of AIS in recent years [41]. FE biomechanical analysis can establish a similar model to simulate the biomechanical response of corresponding structures in the process of disease progression, or treatment based on the preoperative medical image data of patients, to find the cause and optimize a treatment strategy. In 2019, Wang et al. [42] proposed an FE method to explore the effect of osteoporosis on internal fixation after spinal osteotomy. Taleghani et al. [43]. used the FE method to explore the effect of IVD implants on scoliosis, which cannot be done in vitro. In this study, the FE method was used to explore the effect of ligament relaxation on the biomechanics of scoliosis lumbar vertebrae, to explore whether the uneven stress distribution of vertebrae can be alleviated by relaxing the ITL on the concave side and to better understand the mechanical characteristics of scoliosis lumbar vertebrae.

AIS can have a huge impact on the shape of the spine, especially in children, which can lead to untreatable spinal malformations or thoracic insufficiency syndrome [44]. The treatment of scoliosis involves surgical instruments and support and is an effective treatment for AIS [7]. A growth rod can effectively give support to the scoliosis spine and prevent further deterioration [45] while allowing the spine grow [46] and some of these implants can reduce deformities by passive sliding [47,48], but the operation is expensive and complex. The most significant characteristic of stress in AIS is uneven distribution. Although it is impossible to determine the causal relationship between uneven stress distribution and scoliosis, it is worth studying whether scoliosis can be improved by relaxing the symptoms of uneven stress distribution in the concave-side ITL. According to Wolf’s law, the vertebral body will make corresponding changes according to pressure to adapt to external changes [43]. The spine is an important part of the human under pressure, and its growth and development are related to external pressure [49]. When the stress of the vertebrae is concentrated on one side, it will inhibit the growth of bone and lead to the abnormal development of the bone. When the stress disappears, the bone will continue [50]. Goto et al. [51] found that spine growth changes with the change of load. This study found that scoliosis changed with the load. It only affects the bending load, under which the maximum stress of the IVD and vertebral body will increase, especially when bending to the convex side, it increases stress on the convex side of the vertebral body, especially the stress of the IVD, and increases the risk of IVD injury.

After relaxation of ITL, the ROM of the joint under lateral bending load is increased, and the angle of motion of AIS lumbar vertebrae increases, which narrows the intervertebral foramen, whereas the lateral pressure of the IVD increases. As the stability of the lumbar vertebra decreases, the stress on one side of intervertebral disc increases, and it is easy to oppress the nerves around the intervertebral foramen [52] leading to related lesions [53], for example, intermittent low back pain or leg pain caused by nerve impact [54]. Therefore, the instability of the lumbar spine caused by relaxation of ITL on the concave side will further induce related neurological diseases [24].

Kamal et al. [21] used an FE model of musculoskeletal mixture. Their simulation results show that the weakening of concave-side multifidus lumborum muscles and longissimus thoracis pars thoracic can lead to the correction of axial rotation of AIS spine and the sagittal symmetrical distribution of more uniform von-Mise stress in the endplate. By reducing the strength of longissimus thoracis pars thoracic and multifidus lumborum muscles on the concave side, thereby reducing compressive stress on the concave side of the vertebrae, the progression of scoliosis will be reduced [8]. This study only relaxed the stiffness of the ITL on the concave side and did not consider the muscle on the scoliosis. This study found that after the relaxation of the ITL on the concave side, under six kinds of loads, it only affected the stress of the vertebrae under the bending load but did not reduce stress on the concave side of the vertebral body. Therefore, the relaxation of ITL on the concave side alone will reduce the stability of the lumbar and increase stress on the concave side of the vertebrae. Relaxing the stiffness of the ITL on the concave side alone cannot treat scoliosis and will aggravate the progress of scoliosis.

The FE method can be used to explore the biomechanical properties of spine [55], but this study also has some limitations. In this study, we only explored the lumbar spine but did not explore the effect of relaxed ITL on a multi-segmental spine. The static structure was analyzed, and the dynamic growth of the vertebral body was not taken into account. Many AIS patients have a thoracic curve so, in the next study, we will analyze the multi-stage spine. The model established in this study was simplified to a certain extent, without considering the mechanical response under muscle load. There are unavoidable flaws in the establishment of the models in this study thus making them imperfect systems. Imperfection is not always harmful, as it can lead to unexpectedly complex and organized behavior [56]. Only one subject was selected in this study so the scoliosis type may influence the results.

## 5. Conclusions

An effective scoliosis lumbar model was established. Laxity of the ITL on the concave side of the scoliotic lumbar spine only affected the bending load. The laxity of the concave-side ligament reduced the stability of the lumbar, especially the stability of convex scoliosis by 21.4%, and aggravated the uneven stress distribution of the scoliotic lumbar vertebrae. The stress of the lumbar vertebral body increased by 23.4–42.0%, and the stress of IVD increased by 23.3–78.2%, which could lead to an increase in the risk of IVD injury, and be unfavorable for the scoliotic lumbar spine. For the treatment of scoliosis, relaxation of the concave ITL alone is not an effective way to treat scoliosis.

## Figures and Tables

**Table 1:** Material properties and mesh sizes of solid parts.

Component	Yang’s Modulus E (MPa)	Poisson’s Ratio (*v*)	Mesh Size (mm)
Cortical bone	12,000	0.30	0.5
Cancellous bone	100	0.30	1
Endplate	25	0.25	0.5
Annulus fibrosis	4.2	0.45	1
Articular cartilage	50	0.30	0.5
Nucleus pulposus	1	0.50	1

**Table 2:** Material properties of ligaments.

Ligament	Young’s Modulus (MPa)	Cross-Sectional Area (mm^2^)	Average Length (mm)
Anterior longitudinal ligament	7.8	22.4	20
Posterior longitudinal ligament	10.0	7.0	12
Ligamentum flavum	17.0	14.1	15
Interspinous ligament	10.0	0.6	32
Capsular ligament	7.5	10.5	5
Supraspinous ligament	8.0	10.5	11
Intertransverse ligament	10.0	14.1	13
Relaxation of intertransverse ligament	5.0	14.1	13

**Table 3:** Comparison of average stiffness between the models in the study.

Experiment	Average Stiffness (Nm/°)				
	Moment (Nm)	Flexion	Extension	Lateral Bending	Axial Rotation
Yamamoto [33]	10	1.75	3.22	2.44	5.26
Heth [38]	6	1.10	2.35	1.33	2.61
Present study	10	1.51	2.21	2.57	4.87
Percent of differences		13.8%	31.3%	7.0%	7.41%

## Data Availability

The data that support the findings of this study are available on reasonable request from the corresponding author. The date is not publicly available, due to privacy or ethical restrictions.
